# Stress and emotional wellbeing among LGBT older adults: a moderated mediation model for social network size, composition, and perceived social support

**DOI:** 10.1093/geront/gnaf198

**Published:** 2025-09-11

**Authors:** Anyah Prasad, Jeffrey A Burr, Edward Alan Miller, Karen Fredriksen-Goldsen

**Affiliations:** Department of Medicine, Health, and Society, Vanderbilt University, Nashville, Tennessee, United States; Department of Gerontology, University of Massachusetts Boston, Boston, Massachusetts, United States; Department of Gerontology, University of Massachusetts Boston, Boston, Massachusetts, United States; Department of Health Services, Policy and Practice, School of Public Health, Brown University, Providence, Rhode Island, United States; School of Social Work, University of Washington, Seattle, Washington, United States

**Keywords:** Homophily, Family of choice, Peer group, Social cohesion, Stress buffering

## Abstract

**Background and Objectives:**

Previous research has not differentiated between the structural and functional dimensions of social networks when trying to understand whether social relationships have a moderating or mediating role in protecting against the effects of stress on health. This study investigates if social network size and composition moderate the mediation role of perceived social support between stress and emotional wellbeing.

**Research Design and Methods:**

Among a national sample of LGBT Americans aged 50 years and above, we investigated the effect of a single moderator (total social network size) and two pairs of moderators separately (LGBT and non-LGBT social network size; size of social network members ≥50 years old and <50 years old) on the direct and indirect paths between stress and emotional wellbeing (depressive symptoms, loneliness), the indirect path being mediated by perceived social support.

**Results:**

Diminishing perceived social support partially mediated the association between stress and emotional wellbeing. Total social network size moderated both the direct and indirect association between stress and depressive symptoms but only moderated the indirect association between stress and loneliness. When social network size was grouped by LGBT identity and age, only social networks composed of LGBT and older members demonstrated moderation effects.

**Discussion and Implications:**

With relatable life experiences, LGBT older adults may feel more supported by similar others in their social networks in protecting against the negative association between stress and emotional wellbeing. These findings offer insights for the development of social network interventions to promote LGBT older adults’ health.

## Background and objectives

Depression is one of the most prevalent mental health conditions and is often concomitant with feelings of loneliness (Jeste et al., 2020). Both mental health and social connections play a vital role in shaping the overall emotional wellbeing of older adults. Among a national sample of LGBT (Lesbian, Gay, Bisexual and Transgender) older adults, a health disparate population, nearly one-third screened positive for depression (Fredriksen-Goldsen et al., 2011), significantly higher than their heterosexual peers (Dai & Meyer, 2019; Rowan et al., 2022). LGBT older adults are also at risk of social isolation and loneliness, as they are less likely to be married/partnered or have children and likely to be estranged from their families of origin (Knauer, 2016). Indeed, about 55% of LGBT older adults reported living alone, and they are significantly lonelier compared to their heterosexual counterparts (Hsieh & Liu, 2021; Kim & Fredriksen-­Goldsen, 2016; Peterson et al., 2023). High rates of depression and loneliness suggest that prejudice toward sexual and gender minorities creates a stressful social environment that compromises LGBT individuals’ emotional wellbeing (Jackson et al., 2019).

The negative association between stress and emotional wellbeing is usually weaker among people with strong social support networks (Sheffler & Sachs-Ericsson, 2016). However, whether social relationships play a moderating or mediating role in protecting against the effect of stress on emotional health is not clear. This lack of clarity is due, in part, to past studies using the structural (size and composition) and functional (perceived social support) aspects of social networks as interchangeable measures of social relationships, which although related, are nevertheless distinct concepts. The current study investigates whether social network size and composition moderate the mediating role of perceived social support in the relationship between stress and emotional wellbeing among LGBT older adults.

### Stress and emotional wellbeing

Although stress is a natural response to challenging situations, chronic stress increases allostatic load by disrupting neuroendocrine homeostasis, causing emotional dysregulation (Andreescu et al., 2019). Aging can be particularly stressful due to the increased risk of health deterioration, financial constraints, caregiving responsibilities, and the death of family and friends. These stressful life events are consistently associated with depression among older adults (Gameiro et al., 2014). Similarly, stress due to discriminatory experiences may increase depressive symptoms among older adults (Ayalon & Gum, 2011). Feeling connected with other people is an essential aspect of socioemotional wellbeing, but a sense of loneliness may be a negative response to stress (Campagne, 2019).

In addition to the stressors experienced by older adults generally, LGBT older adults are often exposed to “minority stress” (Hatzenbuehler, 2009; Meyer, 2003). The stressors specific to LGBT identity may result from discrimination and identity concealment that are associated with depression among LGBT older adults (Fredriksen-Goldsen et al., 2017; Hoy-Ellis & Fredriksen-Goldsen, 2016). Further, the stress caused by marginalization may lead to loneliness among LGBT persons by activating internalized homonegativity and social inhibition (Elmer et al., 2022; Kuyper & Fokkema, 2010). The stress from discrimination may also negatively impact same-sex relationships (Rostosky & Riggle, 2017), potentially causing alienation and loneliness. Further, heightened stress due to their intersectional marginalization may explain some of the worse emotional health outcomes among subgroups of LGBT older adults (Kim, Jen, et al., 2017).

### Stress and emotional wellbeing: role of social networks

People embedded in larger social networks and those who perceive better social support availability are more likely to experience better emotional wellbeing (Gariépy et al., 2016; Santini et al., 2015). While social relationships may directly influence emotional wellbeing, these relationships may also be protective by cushioning the negative effect of discrimination and marginalization on emotional wellbeing. Studies demonstrate that social network size moderates the association between discrimination and emotional wellbeing, such that LGBT older adults, with larger social networks, report better emotional wellbeing in the face of discrimination (Leahy & Chopik, 2020). Although social network composition, another structural aspect of social networks, is also associated with emotional wellbeing (Kim, Fredriksen-Goldsen et al. 2017), its role as a stress buffer has not been explored for LGBT older adults.

There is often a strong, positive correlation between social network size and perceived social support (Wang, 2016). Indeed, older adults with larger social networks perceive significantly higher levels of social support (Grossman et al., 2000). Therefore, it was initially hypothesized that perceived social support, like social network size, might moderate the association between discrimination and emotional wellbeing. However, empirical evidence for the moderating role of perceived social support is sparse. This is reflected in a systematic review, where most studies did not support the moderating role of social support on the association between discrimination and mental health (Schmitt et al., 2014). Instead, Kondrat et al. (2018) argue and demonstrate that perceived social support may mediate and reduce the impact of stress on emotional wellbeing.

The mechanisms by which social network size and perceived social support influence the association between stress and emotional wellbeing may be different. Perceived social support, as an operational aspect of forming and sustaining a social tie, may have a more direct mediating role in coping with stress, helping to maintain emotional wellbeing. By contrast, the structure of social networks (size and composition) may have a more moderating role on the association between stress and emotional wellbeing since social support is harnessed from social networks. In sum, an important conceptual distinction exists between the structural and functional aspects of social relationships, but how these dimensions of social networks are interrelated with each other in their association with emotional wellbeing remains unclear. This is especially the case regarding the potential modulating role network members might play regarding the influence of stress on emotional wellbeing among LGBT older adults.

### Theoretical perspective

The Stress Process Model (Pearlin, 1999) and the Minority Stress Model (Hatzenbuehler, 2009). postulate that stress negatively impacts mental health, but social relationships buffer the adverse effects of stressors on mental health. Thoits (2011) downplays the buffering/moderating role of social support (perceived or received) on health while underscoring the direct mediating role social support may play between stress and emotional wellbeing. Kondrat et al. (2018, p. 307) implore scholars to be more critical of the predominant stress-buffering role of social relationships, stating “How social support is measured may determine if social support operates as a mediator (perceived social support) or moderator (number of social supports).” The possibility that the mediating role of perceived social support between stress and emotional wellbeing may be moderated by social network size and composition requires additional investigation.

Like social network size, social network composition may also exert a moderating effect on the relationship between stress and emotional wellbeing. According to the Homophily Principle (McPherson et al., 2001), social networks are typically composed of similar others, and minority individuals may feel more support when surrounded by social network members with a shared identity (McGaskey et al., 2016; Merino, 2014). LGBT older adults’ social networks are usually friend-centered (Fredriksen-Goldsen et al., 2017). They report feeling more supported by friends than family (Boggs et al., 2017), and their support providers tend to be of the same sexuality (Frost et al., 2016). Furthermore, LGBT older adults may rely more on their age peers for social support due to age segregation in social spaces and limited social interaction among LGBT persons of different generations (Boggs et al., 2017). Hence, the moderating role of social network size may be stronger for LGBT older adults when their social networks are composed of LGBT persons and age peers.

### The current study

**Figure 1.**
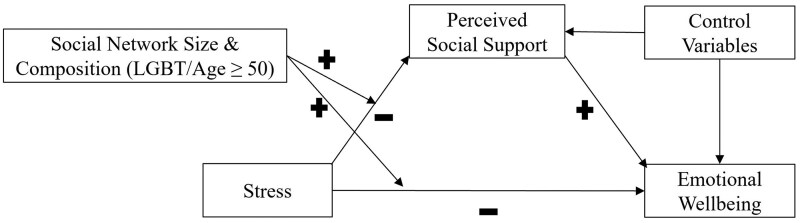
Conceptual model for the moderated mediation role of social network size, composition, and perceived social support on emotional wellbeing.

Based on the empirical evidence and theoretical frameworks discussed earlier, this study examines if perceived social support mediates the relationship between stress and emotional wellbeing (Research Question 1). This study also investigates whether the mediating role of perceived social support on the association between stress and emotional wellbeing is moderated by the size and composition of LGBT older adults’ social networks (Research Question 2, see also Figure 1). We hypothesize that stress, in addition to having a direct effect, will have an indirect negative association with emotional wellbeing by eroding LGBT older adults’ perceived availability of social support. However, those LGBT older adults with larger social networks will continue to feel more supported and better protected from the adverse effects of stress on their emotional wellbeing. Finally, we hypothesize that the stress-buffering effects will be stronger for LGBT and older network members than non-LGBT and younger network members.

Other factors that may confound the relationship between stress, social network characteristics, and emotional wellbeing are included in the analysis. These covariates include education, employment status, income, and marital status, which have been found to be associated with mental health (Silva et al., 2016). There may also be group differences based on age and race/ethnicity (Thomas Tobin et al., 2022). Physical health is another determinant of mental health (Katon, 2011) included in the models.

## Research design and methods

### Data source and study sample

To test the hypotheses, we used data from the first wave of the *Aging with Pride: National Health, Aging, and Sexuality/Gender Study* (NHAS), this is the only wave available for public use. The NHAS is a survey of LGBT Americans aged 50 years and above, based on a purposive sample design with respondents recruited from 11 community-based agencies from across the United States, with some agencies serving older adults irrespective of LGBT identity and some specifically serving LGBT older adults (Fredriksen-Goldsen & Kim, 2017). Between June and November 2010, the questionnaire was mailed or sent electronically by the participating agency to their clientele, along with an invitation letter. A reminder was sent 2 weeks after the initial contact, and a second reminder was sent 2 weeks after the first reminder. Approximately 63% of the surveys were returned that met the inclusion criteria. The number of returned paper and electronic questionnaires from LGBT responders was 2,201 and 359, respectively (*N* = 2,560). After applying listwise deletion to handle missing data, the final study sample size included 2,109 respondents. There were no significant differences in the primary variables of interest between respondents with complete data and those excluded from the analyses due to missing data (Supplementary Table 1). The study procedures of NHAS were approved by the University of Washington Seattle Institutional Review Board.

### Measures

#### Emotional wellbeing

This study used two components of emotional wellbeing as outcome variables. Depressive symptoms were measured with the 10-item short form of the Center for Epidemiological Studies Depression Scale (Radloff, 1977). The scale includes positive items, such as “I felt hopeful about the future,” and negative items, such as “I felt that everything I did was an effort.” Respondents were asked to score those items on a scale that indicated how frequently they felt that way over the past week (0 = *<1 day*, 1 = *1–2 days*, 2 = *3–4 days*, 3 = *5–7 days*). Positive items were reverse coded, and the summed scores ranged from 0 to 30. The score was not computed if more than two items were missing. *Loneliness* is measured (Hughes et al., 2004) as an average of three items; “How often do you feel that you lack companionship?”; “How often do you feel left out?”; “How often do you feel isolated from others?” These items are scored on a scale ranging from 1 = *hardly ever* to 3 = *often*, and the scale was set to missing if more than one item was missing (range = 1–3).

*Perceived stress* is the key independent variable (Cohen et al., 1983). The measure of perceived stress included four items, where respondents were asked how often over the last 4 weeks they “felt that they were unable to control the important things in their life”; “felt confident about their ability to handle their personal problems”; “felt that things were going their way”; and “felt difficulties were piling up so high that they could not overcome them.” The negative statements were scored on a scale ranging from 0 = *never* to 4 = *very often*, but the positive items were reverse coded. Respondents’ stress levels were measured as the mean of the four items but set to missing if missing on more than two items (range = 0–4).

*Perceived social support* is the mediator variable and is measured with the mean of four items that assess respondents’ perception of how frequently someone would be available to provide them with various forms of social support (Sherbourne & Stewart, 1991). The four items were: “Someone to help with daily chores if you were sick”; “Someone to turn to for suggestions about how to deal with a personal problem”; “Someone to do something enjoyable with”; and “Someone to love and make you feel wanted.” Each item was scored on a 4-point scale (1 = *never*, 2 = *seldom*, 3 = *usually*, 4 = *always*) (range = 1–4). The measure was set to missing if data were missing on more than two items.

*Social network size and LGBT/age composition* are moderator variables and were assessed by asking respondents how many people (e.g., friends, family members, colleagues, and neighbors) they interacted with in a typical month (including talking, visiting, and exchanging phone calls or emails). Respondents were also asked to differentiate the number of people they knew by gender, sexuality, and age. Network size overall and by subgroups (LGBT *vs* non-LGBT; age 50 and above *vs* below age 50) was summed. These measures were then grouped into quartiles to account for extreme outliers (range = 1–4).

#### Control variables

Respondent *age* is measured in years; however, to ensure confidentiality, respondents who were 80 years and older were top-coded as 80 years or older by the survey research team (range = 50–80+). *Race and ethnicity* of the respondents are coded as a set of dichotomous variables—non-Hispanic White (reference), non-Hispanic Black, non-Hispanic other race groups, and Hispanic (any race). *Education* is coded as a dichotomous variable (1 = *high school or less*, 0 = *some college or more*). *Income* is specified as a set of dichotomous variables: less than $20,00 (reference); $20,000–$24,999; $25,000–$34,999; $35,000–$49,999; $50,000–$74,999; and more than $75,000. *Employment* status is measured as a dichotomous variable (1 = *employed*, 0 = *not employed*). *Marital/partnership status* is measured as a dichotomous variable (1 = *married or partnered*; 0 = *not married or partnered*). *Physical health* is based on a subjective assessment of respondents’ physical health over the past 4 weeks, measured as a mean of four items from the *SF-8 Health Survey*. Although these items were originally scored either on a five- or six-point scale, both were recalibrated on a scale ranging from 0 to 100, a higher score indicating better physical health. Examples of the two types of items included “How much bodily pain have you had?” (assessed on a Likert scale 1 = *none*, 2 = *very mild*, 3 = *mild*, 4 = *moderate*, 5 = *severe*, 6 = *very severe*; recoded to 1 = 100, 2 = 80, 3 = 60, 4 = 40, 5 = 20, 6 = 0); “How much did physical health problems limit your usual physical activities such as walking or climbing stairs?” (assessed on a Likert scale 1 = *not at all*, 2 = *very little*, 3 = *somewhat*, 4 = *quite a lot*, 5 = *could not do*; recoded to 1 = 100, 2 = 75, 3 = 50, 4 = 25, 5 = 0).

### Analytic strategy

Descriptive statistics for the study sample are presented, followed by bivariate associations between the two measures of emotional wellbeing and stress, social network size and composition, perceived social support, and the control variables. Data preparation, descriptive statistics, and bivariate and regression analyses were performed using SPSS version 27 (SPSS Inc., Chicago, IL). Hayes’ (2013) PROCESS Macro, a software package was used for analyzing if social network size (total, LGBT *vs* non-LGBT, ≥50 *vs* <50 years) moderated the mediating role of perceived social support between stress and emotional wellbeing among LGBT older adults.

The test of significance for the individual paths and their interaction effects is based on *p*-values less than .05. The PROCESS Macro uses 5,000 bootstrapped samples to generate bias corrected 95% confidence intervals for the conditional indirect effects, a product of two individual paths (Hayes, 2013). Standard errors generated by bootstrapping are robust against nonnormality of the dependent variables. PROCESS Macro models allowed us to generate an index of moderated mediation (IMM), along with 95% bootstrapped confidence intervals (BCI), a test of the significance of the moderation effects for the mediation effects (Hayes, 2013).

The models were estimated separately for the number of depressive symptoms and loneliness. Since the overlap between missingness on the two outcome variables was not uniform, descriptive statistics were reported for the 2,109 respondents who had complete data on all the predictors and control variables but may be missing on the number of depressive symptoms or loneliness. The complete sample sizes were 2,060 and 2,104 for the depressive symptoms and loneliness models, respectively.

## Results

Descriptive characteristics of the study sample are reported in Table 1. Respondents’ mean level of depressive symptoms was 7.34 (range = 0–30). Respondents’ mean loneliness was beyond the scale’s midpoint (1.74, range = 1–3), and perceived stress was relatively low (1.23, range = 0–4). Based on a scale ranging from never to always, respondents, on average, are likely to perceive that social support is usually available (3.11), when needed. Respondents had an average of 64 members in their social networks, about half were LGBT and a little more than half were age-peers (social network quartiles are reported in Table 1). Respondents, on average, were 66 years old and mostly non-Hispanic white, where a majority of respondents had more than a high school education, were concentrated more in the middle- and higher-income groups, and scored reasonably high on physical health. About 45% of the respondents were employed and either married or partnered.

**Table 1.** Descriptive characteristics of the study sample and bivariate associations between social network size and LGBT–age composition, perceived social support, stress, emotional wellbeing, and the control variables

	Mean/%	*SD*	Range	Bivariate associations with	
				DS	Loneliness
**Depressive symptoms**	7.34	6.35	0–30		
**Loneliness**	1.74	0.66	1–3		
**Stress**	1.23	0.80	0–4	0.73***(*r*)	0.49***(*r*)
**Social network size—overall**	2.51	1.11	1–4	−0.21***(*r*)	−0.25***(*r*)
** LGBT social network size**	2.52	1.11	1–4	−0.18***(*r*)	−0.23***(*r*)
** Non-LGBT social network size**	2.53	1.09	1–4	−0.19***(*r*)	−0.21***(*r*)
** ≥50 Social network size**	2.53	1.09	1–4	−0.19***(*r*)	−0.26***(*r*)
** <50 Social network size**	2.55	1.09	1–4	−0.16***(*r*)	−0.18***(*r*)
**Perceived social support**	3.11	0.78	1–4	−0.41***(*r*)	−0.62***(*r*)
**Age**	65.74	8.26	50–80	−0.06** (*r*)	−0.07*** (*r*)
**Race/Ethnicity**				4.78**(*F*)	1.61(*F*)
** Non-Hispanic White**	87.5%		1–4		
** Non-Hispanic Black**	3.2%				
** Non-Hispanic other**	5.4%				
** Hispanic**	3.9%				
**Income**			1–6	44.86***(*F*)	52.01***(*F*)
** Less than $20,000**	16.4%				
** $20,000–$24,999**	7.7%				
** $25,000–$34,999**	11.9%				
** $35,000–$49,999**	14.4%				
** $50,000–$74,999**	17.5%				
** More than $75,000**	32.1%				
**Education (1 = high school or less)**	7.0%		0–1	3.10(*F*)	4.20*(*F*)
**Married/partnered**	44.8%		0–1	24.09***(*F*)	46.13***(*F*)
**Employed**	45.3%		0–1	27.50***(*F*)	7.06**(*F*)
**Physical health**	70.12	22.17	5–100	−0.48***(*r*)	−0.26***(*r*)

*Note*. *N = *2,060 for depressive symptoms, 2,104 for loneliness, and 2,109 for all other variables. Bivariate associations were estimated with Pearson correlation for continuous by continuous variables; *t*-test or analysis of variance for continuous by categorical variables; chi-square test for categorical by categorical variables; DS = the number of depressive symptoms.

* *p* < .05.

** *p* < .01.

*** *p* < .001.

For the bivariate associations (Table 1), perceived stress was strongly correlated with the number of depressive symptoms (*r *= 0.73) and moderately correlated with loneliness (*r *= 0.49). Total social network size and network size defined by LGBT identity and age group, as well as perceived social support, were negatively correlated with depressive symptoms and feelings of loneliness. All the control variables showed significant bivariate associations with the two outcome variables, except number of depressive symptoms and education, and loneliness and race–ethnicity.

**Figure 2.**
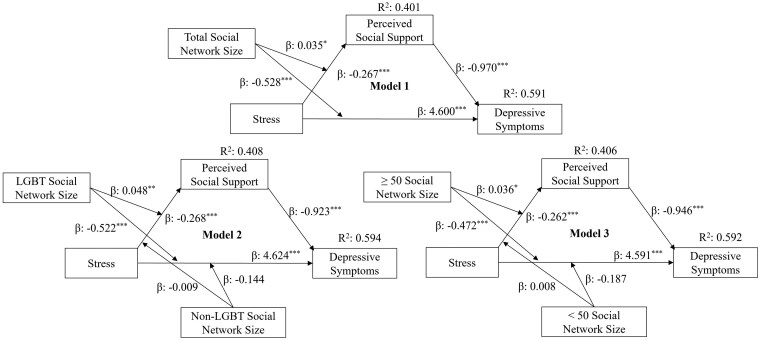
Moderation effects of social network size and LGBT–age composition on direct and indirect pathways for the association between stress and the number of depressive symptoms. ^*^p < .05. ^**^p < .01. ^***^p < .001.

**Figure 3.**
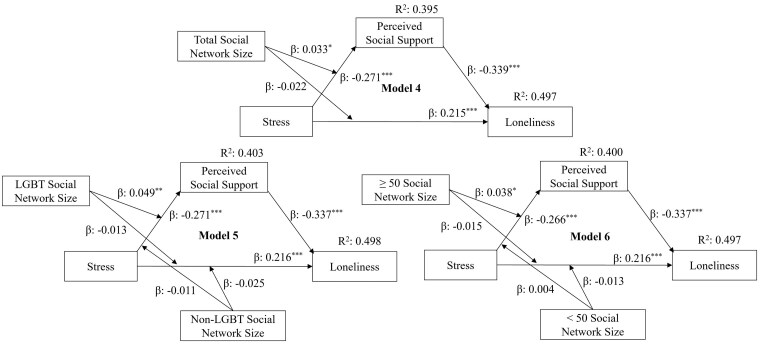
Moderation effects of social network size and LGBT–age composition on direct and indirect pathways for the association between stress and loneliness. ^*^p < .05. ^**^p < .01. ^***^p < .001.

The individual regression paths between perceived stress, perceived social support, and emotional wellbeing, along with the moderation effects of social network sizes of the individual paths are presented in Figures 2 and 3. Along with coefficients for the direct paths and their interaction terms, the coefficients of the indirect paths (a product of the two individual paths: perceived stress—perceived social support; perceived social support—emotional wellbeing) and their IMM are presented in Table 2.

**Table 2.** Indirect effects of stress on depressive symptoms and loneliness through perceived social support and the moderation effects of social network size and composition

Model	Path Moderator/s	Direct effect Moderation effect	Indirect effect (BCI) Index of moderated mediation (BCI)
**1**	Stress—>PSS—>DS	4.600***	0.259 (0.168, 0.359)
	Total SNS	−0.528***	−0.034 (−0.070, −0.004)
**2**	Stress—>PSS—>DS	4.624***	0.248 (0.158, 0.347)
	LGBT SNS	−0.522***	−0.044 (−0.086, − 0.010)
	Non-LGBT SNS	−0.144	0.009 (−0.025, 0.046)
**3**	Stress—>PSS—>DS	4.591***	0.248 (0.160, 0.343)
	≥50 SNS	−0.472***	−0.034 (−0.075, −0.002)
	<50 SNS	−0.187	−0.007 (−0.040, 0.027)
**4**	Stress—>PSS—>Loneliness	0.215***	0.092 (0.075, 0.0110)
	Total SNS	−0.022	−0.011 (−0.022, −0.001)
**5**	Stress—>PSS—>Loneliness	0.216***	0.092 (0.075, 0.110)
	LGBT SNS	−0.013	−0.017 (−0.029, −0.005)
	Non-LGBT SNS	−0.025	0.004 (−0.008, 0.016)
**6**	Stress—>PSS—>Loneliness	0.216***	0.090 (0.073, 0.107)
	≥50 SNS	−0.015	−0.013 (−0.025, −0.001)
	<50 SNS	−0.013	−0.001 (−0.013, 0.011)

*Note. N *= 2,060 for depressive symptom models, 2,104 for loneliness models; SNS = social network size; PSS = perceived social support; DS = depressive symptoms; BCI = bootstrapped confidence intervals.

* *p* < .05.

** *p* < .01.

*** *p* < .001.

In Models 1 and 4 (see Table 2), stress had a statistically significant direct association (β = 4.600, *p* < .001; β = 0.215, *p* < .001, respectively) and indirect association (β = 0.259, BCI 0.168, 0.359; β = 0.092, BCI 0.075, 0.011, respectively) with number of depressive symptoms and loneliness, mediated by perceived social support. Total social network size moderated both the direct association (β = −0.528, *p* < .001) and indirect association (β = −0.034, BCI −0.070, −0.004) between stress and the number of depressive symptoms but moderated only the indirect association between stress and loneliness (β = −0.011, BCI −0.022, −0.001). In Models 2 and 5, when social network size was grouped by LGBT and non-LGBT status, LGBT social network size moderated the direct association (β = −0.522, *p* < .001) and indirect association (β = −0.044, BCI −0.086, −0.010) between stress and the number of depressive symptoms and the indirect association between stress and loneliness (β = −0.017, BCI −0.029, −0.005). Non-LGBT social network size did not show any moderation effect. Similarly, in Models 3 and 6, where social networks were defined by age group, the size of the 50+ age social network group moderated the direct association (β = −0.472, *p* < .001) and indirect association (β = −0.034, BCI −0.075, −0.002) between stress and the number of depressive symptoms and the indirect association between stress and loneliness (β = −0.013, BCI −0.025, −0.001). However, the social network size of the age group less than 50 years old showed no moderation effect. About 40% of the variation in perceived social support, 59% of the variation in the number of depressive symptoms, and 50% of the variation in loneliness were accounted for in the models.

## Discussion and implications

There is wide wide-ranging consensus in the scientific literature that social relationships not only foster human health and wellbeing but also protect against the ill effects of stressors in life (Santini et al., 2015; Umberson & Karas Montez, 2010). However, the distinction between the structural and functional features of social networks and their potential buffering mechanisms for the relationship between stress and emotional health is not well-studied, especially among LGBT older adults. As predicted by the Stress Process Model and Minority Stress Model (Hatzenbuehler, 2009; Pearlin,1999), the results from this study showed that stress had a significant positive association with depressive symptomatology and feelings of loneliness among LGBT older adults, where social network characteristics weakened the association. Stress had adverse association with emotional wellbeing through reducing perceived social support, but the strength of this mediation was inversely proportional to LGBT older adults’ social network size, supporting Thoits (2011) and Kondrat et al. (2018). Also, as related to the Homophily Principle (McPherson et al., 2001), the strength of the moderation of the mediation was stronger when LGBT older adults’ social networks were composed of other LGBT persons and age peers.

Feeling supported by people in one’s social network often instills a sense of confidence that there will be resources to fall back on in times of need. The perceived availability of social support encourages people to explore and take risks and to strive for the best of their potential in many aspects of their lives. The increased confidence that comes from a perception of available social support may strengthen people’s psychological resources, including sense of self-control, self-esteem, and self-worth, which can directly contribute to their emotional wellbeing (Thoits, 2011). However, stressors, such as discrimination or other challenges that arise with aging, may be overwhelming, eroding confidence about potential support, especially if surrounded by fewer social network members, thereby adversely impacting emotional wellbeing.

LGBT older adults may feel the need for support in dealing with stressful situations and want to activate actual support. Having people in their lives with whom they can talk and receive advice from may help LGBT older adults cope with stressful events, safeguarding their emotional wellbeing. Having more people in their social network means more opportunities for support. Therefore, social network size, as a potential reserve of resources, acts as a moderator in mitigating the adverse effects of stress on emotional well-being. This is in contrast with perceived social support which, as an indicator of the ability to tap into resources while facing stress, is better operationalized as a mediator in the stress–emotional wellbeing relationship.

Findings from this study suggested that the network composition defined by LGBT identity and age group is other aspects of social network structure that condition the mediating role of perceived social support. It may be that LGBT older adults feel that only other sexual and gender minority peers understand the full gravity of dealing with such stressors as shame, stigma, discrimination, or aging with less traditional social ties. Age peers, having lived through discriminatory policies of the past and the HIV crisis, may be better able to understand and support LGBT older adults (Boggs et al., 2017). As a generation that faced social injustices, LGBT older adults likely experienced a greater sense of group cohesion with their LGBT and age peers compared to non-LGBT and younger network members (Willis et al., 2018). If there are more LGBT persons and more older adults in their social networks, LGBT older adults may be more confident about the availability of appropriate social support needed to face prevailing stressors. Our results are parallel with the results from other studies showing that LGBT older adults with more friends in their social networks, usually composed of LGBT age peers, felt more supported and had better emotional health (Brennan-Ing et al., 2017; Grossman et al., 2000). Comparable observations about the relevance and salience of social support from homophilous network members have been made among other marginalized groups, such as racial/ethnic minorities and religious minorities (McGaskey et al., 2016; Merino, 2014). For instance, the cognitive and emotional burden of evaluating whether an individual comprehends racial discrimination and can be deemed trustworthy is often alleviated in interactions with those who share one’s racial or cultural identity (To et al., 2020).

Overall social network size and networks composed of LGBT persons and older peers moderated both the direct and indirect association between stress and depressive symptomatology. However, these characteristics only moderated the indirect association between stress and loneliness. This is probably because feeling lonely is more of an interpersonal experience than depression. In this case, social support in the form of companionship and emotional support may play a more active role in preventing LGBT older adults from withdrawing from their social networks as a response to stress and slipping into social isolation and loneliness. Therefore, the mediating role of perceived social support between stress and emotional wellbeing is stronger when LGBT older adults were embedded in larger and more homophilous social networks.

## Limitations

The cross-sectional research design of this study captured a snapshot in time, limiting the ability to rule out reverse causality, as well as limiting full evaluation of mediation effects. It is possible that feelings of depression and loneliness may induce stress or cause LGBT older adults to withdraw from their social networks, becoming more pessimistic about the availability of social support. Additionally, measurement bias may be present. Future studies may use real-time monitoring of heart rate and blood pressure or biomarkers, such as C-reactive protein instead of perceived stress, which may be subject to recall or social desirability bias. In addition to the structural and functional aspects, relationship quality (positive, negative, and ambivalent) is another dimension of social networks that is vital for perceived social support but this was beyond the scope of the present study (Holt-Lunstad, 2018). Also, as the data in this study were not based on a probability sample, we cannot be confident of the generalizability of our study results to the population of LGBT older adults. Further, past studies documented that health disparities among racial/ethnic minority LGBT older adults may be mediated by lower perceived social support (Kim, Jen, et al., 2017). This study did not consider the homophily of social networks based on other central social characteristics such as race/ethnicity or intersectional identities and their association with perceived social support and emotional wellbeing.

## Implications for research, policy, and practice

Future research should differentiate between the structural and functional aspects of social networks and employ longitudinal data to better understand the stress process model of social relationships and emotional wellbeing. To reduce the negative effect of stress, aging service providers should find ways to create opportunities for LGBT older adults to socialize and continue to stay in touch with their social networks, especially other LGBT persons and age peers. Strategies for activating social networks should be developed to help LGBT older adults cope with stress, safeguarding against loneliness and depression, both of which can have further adverse downstream effects (Lépine & Briley, 2011; Stessman et al., 2014).

## Conclusion

As survivors of life-long marginalization and minority stress, the present generation of LGBT older adults is a resilient group (Colpitts & Gahagan, 2016), which is reflected in their ability to form supportive social networks. This study contributed to the scientific literature by generating a more robust understanding of LGBT older adults’ emotional wellbeing using a large, national data source. The study demonstrated the mitigating role of supportive social networks for the negative consequences of stress. Specifically, the results from this study indicated that the structural features of social networks, including size and composition, exerted a moderation effect, and the functional features of social networks, measured as perceived social support, acted as a mediator in the association between stress and emotional wellbeing.

## Supplementary Material

gnaf198_Supplementary_Data

## Data Availability

Analytical methods and material are available upon request. The data used in this study can be obtained by emailing AgePride@uw.edu. The study was not preregistered.
