# Supplementary material for: Stress and emotional wellbeing among LGBT older adults: a moderated mediation model for social network size, composition, and perceived social support
Source: Gerontologist. 2025 Sep 11;65(11):gnaf198. doi: 10.1093/geront/gnaf198 (PMC12543375; doi:10.1093/geront/gnaf198)
Supplement: gnaf198_Supplementary_Data [file gnaf198_supplementary_data.zip › Prasad et al. Suppl.docx]

**Supplementary Material**

**Supplementary Table 1**

*Differences between respondents with complete data and those excluded from the analyses due to missing data.*

|  | Mean/ % | | Significance of the Difference (*p*) |
| --- | --- | --- | --- |
|  | Analytical Sample  (N = 2109) | Excluded Sample  (N = 128-461) |  |
| Perceived social support | 3.10 | 3.03 | 0.091 |
| Social network size | 54.50 | 57.84 | 0.493 |
| LGBT Social Network Size | 29.54 | 32.57 | 0.365 |
| >50 | 18.99 | 22.82 | 0.196 |
| <50 | 11.59 | 12.28 | 0.743 |
| Non-LGBT Social Network Size | 28.94 | 28.83 | 0.997 |
| >50 | 16.97 | 16.47 | 0.841 |
| <50 | 13.80 | 13.83 | 0.992 |
| Social network LGBT composition | 0.56 | 0.56 | 0.885 |
| Age | 65.74 | 67.78 | <0.001 |
| Race/Ethnicity |  |  | 0.006 |
| Non-Hispanic White | 87.5% | 81.7% |  |
| Non-Hispanic Black | 3.2% | 5.0% |  |
| Non-Hispanic Other | 5.4% | 6.6% |  |
| Hispanic | 3.9% | 6.7% |  |
| Income |  |  | <0.001 |
| less than $20,000 | 16.5% | 27.5% |  |
| $20,000 to $24,999 | 7.8% | 10.9% |  |
| $25,000 to $34,999 | 11.8% | 11.4% |  |
| $35,000 to $49,999 | 14.5% | 13.3% |  |
| $50,000 to $74,999 | 17.5% | 14.3% |  |
| more than $75,000 | 32.0% | 22.6% |  |
| Education (1=high school or less) | 7.0% | 12.3% | <0.001 |
| Married/partnered | 44.8% | 41.9% | 0.262 |
| Employed | 45.3% | 37.27% | 0.002 |
| Had children | 24.7% | 24.1% | 0.797 |
| Self-reported health | 4.40 | 4.33 | 0.244 |

*Note*. Differences were calculated with t-test for continuous variables and chi-square test for categorical variables.
